# Low-intensity pulsed ultrasound stimulation in different regions in the viability of myocutaneous flaps

**DOI:** 10.1186/s40349-016-0069-4

**Published:** 2016-10-21

**Authors:** Fernanda Luiza de Almeida Albuquerque, Lais Mara Siqueira das Neves, Elaine Caldeira de Oliveira Guirro

**Affiliations:** Postgraduate Program in Rehabilitation and Functional Performance, University of São Paulo, Avenue Bandeirantes, 3900, Ribeirão Preto, CEP: 14049-900 São Paulo Brazil

**Keywords:** Thermography, Myocutaneous flap, Skin temperature

## Abstract

**Background:**

Low-intensity pulsed ultrasound (LIPUS) has presented good results in the healing of chronic wounds. The objective of this study was to compare the effect of LIPUS on the viability of transverse rectus abdominal muscle (TRAM) flap in different regions (central and epigastric) in rats.

**Methods:**

Twenty-one Wistar male rats were homogeneously distributed into three groups as follows: group 1 (control), animals submitted to surgery only; group 2, animals submitted to surgery and application of LIPUS at the center of the flap; and group 3, animals submitted to surgery and application of LIPUS at the flap area corresponding to the right inferior epigastric artery pedicle. Stimulation was performed immediately after the surgery and within the following 2 days. The percentage of flap necrosis was evaluated by using the ImageJ® software as well as by measuring the temperature variation with infrared thermography (FLIR® T300).

**Results:**

In the percentage calculation of the necrosis area, the application of LIPUS at the center of the flap (group 2) showed significantly smaller difference (26.2 %) compared to group 1 (54.50 %) and group 3 (44.01 %). Analysis of the temperature variation between the groups was performed by using the one-way ANOVA followed by Tukey’s test. The results showed that both forms of LIPUS application showed significant differences compared to the control group.

**Conclusions:**

In view of our results, one can conclude that the application of LIPUS at the center of the flap was effective for the viability of TRAM flap in reducing the necrosis area.

## Background

Silicone implants, deep inferior epigastric perforator, and transverse rectus abdominis myocutaneous flaps are used for reconstruction of the breast tissue. Silicone-gel prosthesis is a popular option which is naturally associated with shorter surgery time and shorter hospital stays as the use of autologous tissues allows more natural and apoptotic breast cells to grow, with the breast tissue becoming less edematous and smoother as the patient ages [1, 2].

The transverse rectus abdominal muscle (TRAM) flap has been widely used for breast reconstruction, but there is a high risk of failure due to necrosis [3], which might increase both the patient’s time at hospital and treatment costs, thus delaying the patient’s return to the daily activities [4].

Several therapeutic resources are indicated in the literature for minimizing the necrosis of flaps, such as transcutaneous nerve electrical stimulation [5], electro-acupuncture [6], light-emitting diode [7], and low-intensity laser [8].

The low-intensity pulsed ultrasound (LIPUS) was developed for consolidation of complex bone fractures by promoting an increase in the migration of osteoblasts [9, 10]. However, the literature has also demonstrated the efficacy of LIPUS in the repair of tissues [11, 12], with the increment of granulation tissue, collagen fibers, and glycosaminoglycans [13–15], neovascularization [16], and increase in cell viability [15]. The technique is approved by Food and Drug Administration (FDA) and CE Mark for wound healing. It is a non-thermal and non-invasive treatment, at low cost, by promoting angiogenesis, adhesion of leukocytes, growth factor and collagen production, and fibrinolysis, increasing macrophage and nitric oxide levels [17].

The incidence of necrosis in TRAM flaps occurs at different levels, affecting the further areas of the epigastric artery where the highest necrosis rates are found [18]. Therefore, it is important to detect the effect of different therapeutic resources on the viability of this type of flap, including the efficiency of stimulation with LIPUS in the different areas of application.

## Methods

### Ethical considerations

This study has been approved by the Animal Research Ethics Committee of the Medical School of Ribeirão Preto, University of São Paulo, Brazil, according to protocol number 058/2014.

### Materials

Twenty-one Wistar male rats weighing 280–300 g were randomly distributed into three groups of seven animals each: group 1 (control), animals submitted to surgery with TRAM flap only; group 2, animals submitted to surgical procedure and application of LIPUS at the center of the flap; and group 3, animals submitted to surgical procedure and application of LIPUS at the area corresponding to the pedicle with irrigation of the right inferior epigastric artery (flap zone 1).

### Surgical procedure

The animals were maintained and housed in individual cages with a 12-h light-darkness cycle and fed with proper food and water ad libitum*.* Prior to the surgical procedure, the animals were anesthetized with intra-peritoneal injection of ketamine (Agener União®) and xylazine (Dopaser®) at concentrations of 0.1 and 0.07 mL/100 g, respectively. Next, the animals were positioned on a surgical board and submitted to hair removal by means of manual traction followed by surgical procedure for obtaining the myocutaneous flap from the transverse rectus abdominal muscle (TRAM) unilaterally and caudally. The flaps measuring 5 cm in the lateral-lateral direction and 3 cm in the cranial-caudal direction were positioned at 1 cm (causal direction) from the xiphoid process [18].

### Low-intensity pulsed ultrasound

The parameters of the device were set to the following values: SATA low intensity 30 mW/cm^2^, 1.5 MHz resonance frequency, pulsed mode with pulse width 200 μs and 1 kHz repetition frequency, circular transducer of 22 mm and piezoelectric element PZT [11]. The ultrasound was developed by the School of Engineering of São Carlos/University of São Paulo.

**Fig. 1:**
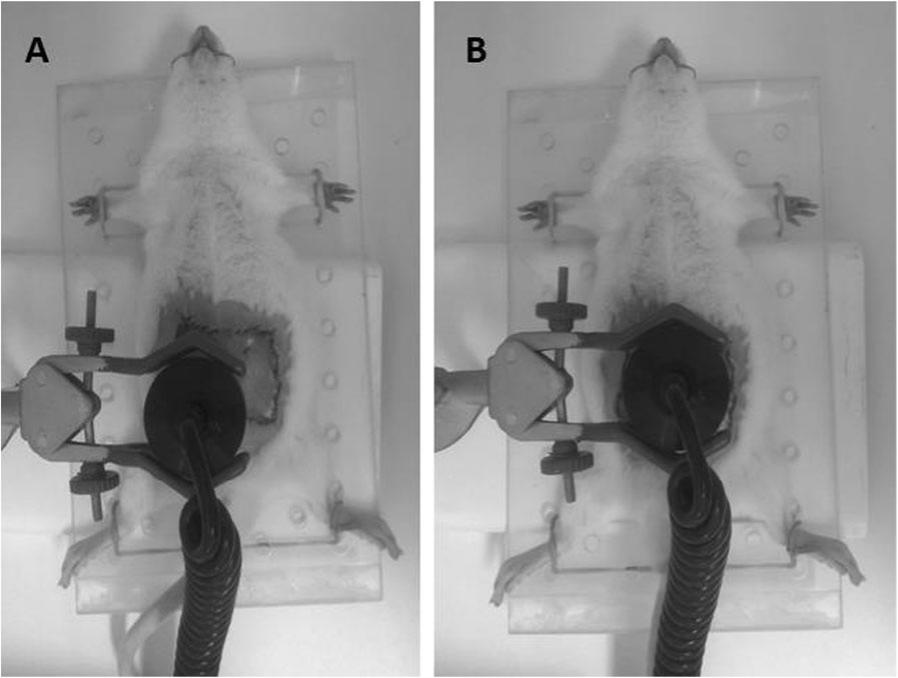
Site of application of the ultrasound transductor. **a** LIPUS positioned at the flap zone 1 (group 3); **b** LIPUS positioned at the center of the flap (group 2)

The transducer was coupled with sterile gel water-based and water-driven equipment through an on/off button. The application of ultrasound was performed once a day for 20 min on a stationary basis immediately after the surgery and within the following 2 days for a total of three applications. Group 2 had the ultrasound transducer positioned at the center of the flap, whereas group 3 had it positioned at the flap zone 1, as shown in Fig. 1.

### Analysis of the necrosis percentage

The percentage of necrosis area was assessed on the fourth day following the surgery by using the ImageJ® software. In order to delimit the area of viable tissue from the necrotic one—the former characterized by a pinkish color and smooth surface and the latter being cold and darkened, a paper template schematized on vegetable paper was used. Calculation was performed by dividing the necrotic flap area by the total area and then by multiplying the result by 100.

### Infrared thermography

**Fig. 2:**
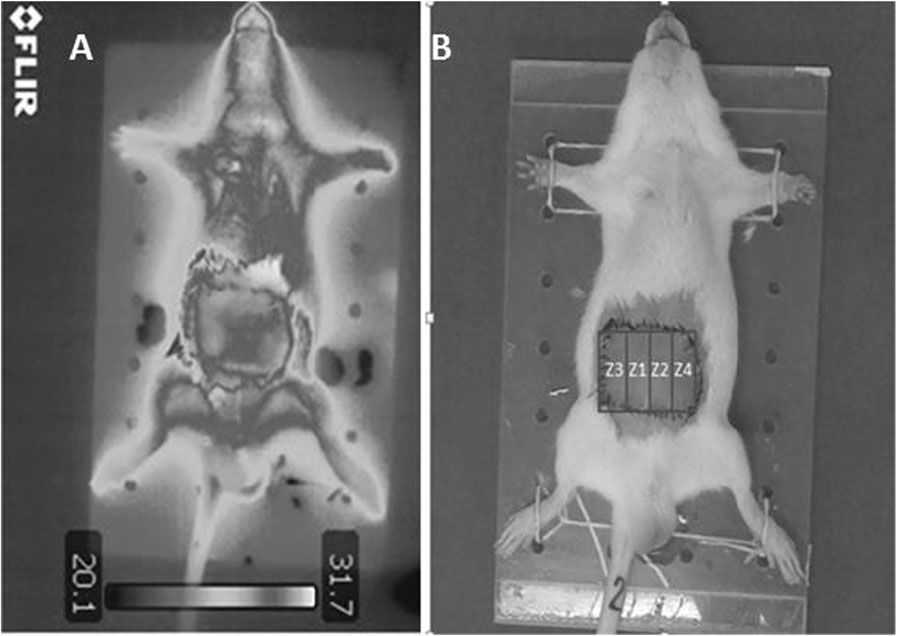
**a** Infrared image of the animal submitted to TRAM flap surgery. **b** The corresponding digital image showing division of the flap into four zones

For analysis of the temperature variation of the flap, images were made on the fourth day following the surgery by using the Flir QuickReport® software. The images were recorded with an infrared camera (T300, FLIR®, Wilsonville, USA). The flaps were analyzed by dividing them into four zones or areas, with zone 1 (Z1) corresponding to the vascular region of the right inferior epigastric artery (Fig. 2). The four zones were of equal size and have the same number of pixels and physical dimension.

Data normality was assessed by using Shapiro Wilk’s test for all variables, whereas analysis of the necrosis percentage and temperature variation between groups was performed by using *one-way* ANOVA and post hoc Tukey’s test. The SPSS version 13.0 software (SPSS Inc. USA) was used for data analysis with a significance level set at 5 %.

## Results

**Fig. 3:**
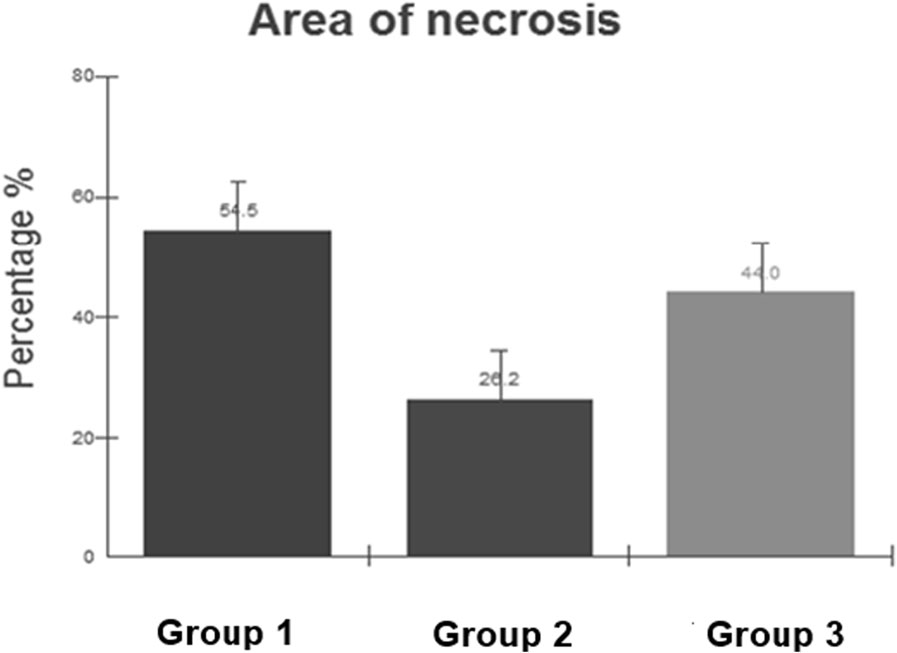
Necrosis area percentage of the experimental groups. Group 1 (control), group 2 (LIPUS applied to the center of the flap), and group 3 (LIPUS applied to the zone 1, where the pedicle is located)

The results found by the use of the ImageJ® software were applied to the formula to calculate the percentage area of flap necrosis. The corresponding values are shown in Fig. 3.

The analysis of necrosis percentage was performed by using one-way ANOVA and post hoc Tukey’s test, demonstrating that there was a significant difference between controls and the group stimulated with ultrasound applied to the center of the flap (*p* < 0.05).

The minimum and maximum temperatures were evaluated, and compared to the maximum temperature, there was no difference between groups, but a significant difference was observed between controls and experimental groups regarding the minimum temperature variation on the fourth day following the surgery (Table 1).

**Table 1:** Values found at minimum temperature (°C) when the groups treated with low-intensity pulsed ultrasound (LIPUS) were compare to the control group. Group 1 is control, group 2 is treated with LIPUS positioned at the center of the flap, and group 3 is treated with LIPUS positioned at the zone corresponding to the epigastric artery, zone 1

	Zone 1	Zone 2	Zone 3	Zone 4
Group 1	29.57*,▪	31.07^α^	30.78	30.07^●,£^
Group 2	27.04*	29.14	28.81	26.25^●^
Group 3	27▪	28.08^α^	28.61	26.7^£^

*p = 0.03 G1 vs G2 (zone 1); ▪*p* = 0.03 G1 vs G3 (zone 1); ^●^ *p* = 0.005 G1 vs G2 (zone 4); ^£^ *p* = 0.01 G1 vs G3 (zone 4); ^α^ *p* = 0.01 G1 vs G3 (zone 2)

## Discussion

The literature points out that the myocutaneous flap is the most popular donor tissue for breast reconstruction in women submitted to mastectomy [1, 18, 19], and therefore, the viability of myocutaneous flaps is of extreme importance for a successful surgery.

The low-intensity pulsed ultrasound stimulation (LIPUS) is a therapeutic resource whose efficacy has been demonstrated in the processes of healing [12, 20], thus being a therapeutic alternative for improving the viability of flaps.

Thermography has been widely used for detection of circulatory alterations by means of temperature [21, 22]. In the present study, analysis of the flap temperature showed that there was a decrease in temperature in both forms of ultrasound application compared to the control group, thus suggesting an efficiency of the resource for this purpose, decreasing the inflammatory process. Other authors [23–25] observed an increase of tissue perfusion, angiogenesis, regional density, and blood flow.

In the present study, the viability of myocutaneous flaps was found to be higher in the group stimulated at the center of the flap compared to that with stimulation of the epigastric artery and controls. These findings may be related to the increase in microcirculation as a result of mechanical waves [25].

The use of LIPUS reduces the damage to cells caused by the applied energy as both fibroblast proliferation and circulatory increment are stimulated, which contributes to cell repair [23]. In addition, LIPUS produces nitric oxide—a gas having a vasodilator action that stimulates the neovascularization as well [25, 26] and whose effect is known to increase the viability of tissues and transplanted organs in cases of ischemia [27]. This might explain the decreased necrosis in the experimental group compared to controls, mainly in the group treated with LIPUS applied to the center of the flap, since centrally applied stimuli favor the formation of new vessels throughout the contour of the flap, consequently making it more viable.

In a study using pluripotent stem cells, artificially induced in culture plates, LIPUS was applied for 7 days and improvement in cell viability was observed soon on the second day of application. The most significant results were found on the fourth day in terms of viability and proliferation of the cells, thus corroborating our study as viability was also observed on the fourth and last days after the surgery [28].

## Conclusions

Based on the results found in the present study, one can conclude that the viability of TRAM flap was higher with the application of low-intensity pulsed ultrasound at the center of the myocutaneous flap.

## Abbreviations

LIPUS: Low-intensity pulsed ultrasound
TRAM: Transverse rectus abdominal muscle
